# Métastase cutanée d'un carcinome indifférencié du cavum: à propos d'un cas

**DOI:** 10.11604/pamj.2020.35.72.16600

**Published:** 2020-03-13

**Authors:** Fadila Kouhen, Fayçal Abbad, Amal Hajjij, Akram Mejdoubi, Nabil Ismaili

**Affiliations:** 1 Department of Radiotherapy, International University Hospital Sheikh Khalifa, Mohammed VI University of Health Sciences, Casablanca, Morocco; 2 Department of Pathology, International University Hospital Sheikh Khalifa, Mohammed VI University of Health Sciences, Casablanca, Morocco; 3 Department of Otorhinolaryngology, International University Hospital Sheikh Khalifa, Mohammed VI University of Health Sciences, Casablanca, Morocco; 4 Department of Medical oncology, International University Hospital Sheikh Khalifa, Mohammed VI University of Health Sciences, Casablanca, Morocco

**Keywords:** Carcinome, nasopharynx, métastase, pronostic, Carcinoma of the nasopharynx, skin metastasis, prognosis

## Abstract

Les métastases cutanées du carcinome indifférencié du cavum représentent une entité clinique rare, de pronostic défavorable. Nous rapportons à travers cette observation le cas d'un jeune patient de 16 ans qui présente 6 mois après la fin de sa radiochimiothérapie concomitante, pour un carcinome indifférencié du cavum, une métastase cutanée unique au niveau du scalp avec une évolution rapide et le patient est décédé un mois après le diagnostic de la métastase. Vu son pronostic sombre, le diagnostic de métastases cutanées doit toujours être évoqué devant des lésions cutanées chez les patients ayant un antécédent de cancer.

## Abstract (en)

Skin metastases from undifferentiated carcinoma of the nasopharynx are a rare clinical entity with unfavorable prognosis. We here report the case of a 16-year-old patient with single scalp metastasis with rapid evolution. This occurred 6 months after the end of concomitant radiotherapy and chemotherapy for the treatment of undifferentiated carcinoma of the nasopharynx. The patient died a month after the diagnosis of the metastasis. Given their poor prognosis, skin metastases should always be suspected in patients with skin lesions after a cancer.

## Introduction

Le cancer du cavum est une entité clinique et histologique qui se distingue des autres cancers de la tête et du cou par son étiologie multifactorielle impliquant le virus Epstein-Barr sans rapport avec l'intoxication alcoolo-tabagique, habituellement rencontrée dans les autres cancers des voies aéro digestives supérieures, sa distribution géographique endémique et sa radio et chimiosensibilité [1]. L'incidence annuelle du cancer du cavum est de 86691 cas (0,6% de tous les cancers) avec plus de 50831 décès chaque année [2]. Les sites métastatiques les plus fréquemment observés sont les ganglions lymphatiques cervicaux, l'os et plus rarement le poumon et le foie. Les métastases cutanées(MC) sont extrêmement rares ne dépassant pas les 1% et sont distinguées par leur pronostic relativement sombre avec une survie médiane ne dépassant pas les 7 mois [3]. Nous rapportons le cas d'un jeune patient de 16 ans qui présente 6 mois après la fin de sa radiochimiothérapie concomitante pour un carcinome indifférencié du cavum, une métastase cutanée unique au niveau du scalp avec une évolution rapide et le patient est décédé un mois après le diagnostic de la métastase.

## Patient et observation

Il s'agit d'un patient de 16 ans non tabagique, sans antécédents pathologiques notables qui s'est présenté au service de radiothérapie en avril 2015 pour une hypoacousie droite, une épistaxis et une adénopathie cervicale droite sans signe neurologique associé. Une biopsie par nasofibroscopie est réalisée et dont l'étude anatomopathologique a objectivé la présence d'un carcinome indifférencié du cavum (type 3 de la classification OMS).

**Figure 1:**
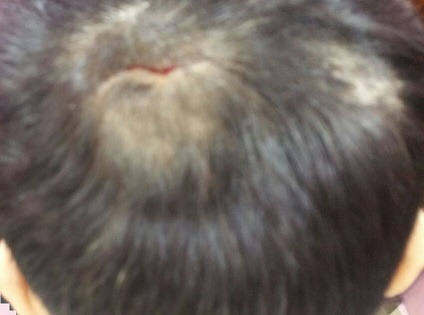
Lésion cutanée au niveau du scalp ulcérée d'environ 3cm

**Figure 2:**
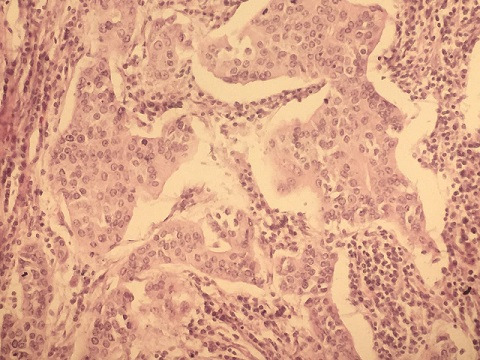
Massif carcinomateux au sein d'un stroma lymphoïde

Une tomodensitométrie (TDM) cervico-thoracique a été demandé dans le cadre du bilan d'extension locorégionale et à distance qui a objectivé un processus tumoral de la paroi postéro-latérale du cavum avec un envahissement de la fosse nasale droite, du sinus sphénoïdal et de la fosse infratemporale, ainsi que la présence des adénopathies jugulo-carotidiennes bilatérales dont la plus grande mesurait 43mm, sans lésion pulmonaire décelable. La scintigraphie osseuse était normale et donc la maladie était classée T4N2M0 selon la classification TNM de l'AJC-UICC de 2009. Le dossier était présenté en réunion de concertation pluridisciplinaire et l'indication d'une radiothérapie externe type arcthérapie à la dose de 70 Gy (2Gy /fraction) et 6 cures de chimiothérapie hebdomadaire à base de cisplatine en concomitant était retenue. Six mois après la fin du traitement, le patient se présente en consultation pour une lésion cutanée unique, non douloureuse qui augmente progressivement de taille au niveau du scalp de 3cm aves des limites irrégulières sans signes inflammatoires en regard (Figure 1). Une biopsie de la lésion était indiquée. L'examen anatomopathologique de la pièce a objectivé la présence d'un massif carcinomateux au sein d'un infiltrat lymphoïde correspondant à une métastase sous galéatiques d'un carcinome indifférencié type UCNT (Figure 2). Une nasofibroscopie réalisée chez le patient était normale. Un bilan d'extension loco-régional et à distance était réalisé à savoir une scintigraphie osseuse et une tomodensitométrie cervico-thoraco-abdominale et qui ont objectivé la présence des lésions d'allure secondaire au niveau osseux (rachis lombaire: L1, L2 et L3) et pulmonaire sans montrer de signe radiologique de récidive locale. Vu le stade métastatique de la maladie, la décision d'une chimiothérapie palliative à base de 5FU et cisplatine était retenue mais le patient est décédé avant le début de traitement.

## Discussion

Nous rapportons un cas exceptionnel d'une métastase cutanée au niveau du cuir chevelue d'un carcinome indifférencié dont l'origine primitive est le carcinome naso-pharyngé traité. Moins de 10 cas ont été rapportés dans la littérature. La métastase cutanée correspond au développement dans le revêtement cutané, d'une tumeur maligne dont l'origine se situe à distance. Elle peut être le premier signe révélateur de l'existence d'un cancer ou le premier signe de récidive métastatique d'un cancer déjà traité [4,5]. La fréquence des métastases cutanées est faible, de l'ordre de 3 à 10% de tous les cancers confondus et dont les cancers les plus pourvoyeurs sont le cancer du sein, du côlon et le cancer pulmonaire [6,7]. Les métastases cutanées des cancers de la sphère ORL, notamment le cancer du cavum, sont extrêmement rares ne dépassant pas les 1% et sont distinguées par leur pronostic relativement sombre [3]. Le mécanisme exact des métastases cutanées est mal connu. Pusieurs hypothèses ont essayé d'expliquer le mécanisme de dissémination et il semblerait que les métastases cutanées à distance du foyer tumoral primitif se propagent via la voie hématogène alors que les métastases locales se propagent par la voie lymphatique dermique ou par manipulation iatrogène à l'origine de l'implantation de cellules tumorales [8,9]. La dissémination hématogène des MC se fait via la valve du système veineux azygos et le système veineux vertébral, connu par le plexus veineux de Batson, contournant le drainage pulmonaire, entrainant ainsi des emboles à tout site drainant vers ce système, y compris le bassin, le cuir chevelu, de la surface du corps. Habituellement, Les patients avec métastases cutanées se présentent avec un stade initial avancé de la maladie (stade III et IV) [10]. Dans le cas rapporté, le cancer du nasopharynx était classé stade III (T4 N2 M0). L'intervalle médian entre le diagnostic initial et la métastase cutanée est de 118 mois. Il était de 6 mois chez notre patient. Le traitement des métastases cutanées n'est pas bien codifié. Devant le pronostic très péjoratif de cette atteinte et compte tenu de la nature métastatique de la maladie, le traitement est, en général, palliatif. Le pronostic des métastases cutanées est souvent sombre avec une survie médiane de 3 à 7 mois et une survie globale de 0% à un an. La survie chez notre patient était d'un mois après le diagnostic de la métastase.

## Conclusion

Les métastases cutanées peuvent être le premier signe révélateur de la rechute métastatique d'un cancer du cavum. Vu son pronostic sombre, le diagnostic de métastases cutanées doit toujours être évoqué devant des lésions cutanées chez les patients ayant un antécédent de cancer.

## Conflits d’intérêts

Les auteurs ne déclarent aucun conflit d'intérêts
